# Beyond Ebola disease: The role of government-citizen relationship in shaping Community outbreak behavior and outcomes

**DOI:** 10.1371/journal.pgph.0007186

**Published:** 2026-08-31

**Authors:** Kasadha Nasser, Klara Fischer, Erika Chenais, Juliet Kiguli, Peter Waiswa, Siya Aggrey, Mats Målqvist

**Affiliations:** 1 Department of Women’s and Children’s Health; Center for Health & Sustainability, Uppsala University, Uppsala, Sweden; 2 Department of Health Policy, Planning and Management, Makerere University School of Public Health, Kampala, Uganda; 3 Department of Urban and Rural Development, Swedish University of Agricultural Sciences, Uppsala, Sweden; 4 Department of Epidemiology, Swedish Veterinary Agency, Uppsala, Sweden; 5 Department of Animal Biosciences, Swedish University of Agricultural Sciences, Uppsala, Sweden; 6 Department of Community Health and Behavioral Sciences, Makerere University School of Public Health, Kampala, Uganda; 7 Uganda Wildlife Research and Training College, Kasese, Uganda; PLOS: Public Library of Science, UNITED STATES OF AMERICA

## Introduction

The ongoing Ebola outbreak in the Democratic Republic of the Congo (DRC), which was recently declared over in Uganda, underscores persistent challenges of preparedness and response to Ebola in complex social, political, and economic environments. Despite medical advances [1] outbreaks recur, exposing critical gaps in the entire mitigation continuum.

Effective outbreak response requires multi-level, multidisciplinary collaboration spanning local, national, and international actors. Collaboration with local communities is key for effective outbreak response. While Uganda has been successful in containing the present and previous Ebola outbreaks, our research shows that disease outbreak management remains dominated by a top-down response logic. Our ongoing work on Ebola outbreak response in western and central Uganda shows how this leads to a lack of trust in government-led interventions and, by extension, to suboptimal compliance. Previous research has reported similar findings from DRC [2–5].

During the current outbreak, as during previous ones, local communities in Uganda and DRC have been documented to label Ebola outbreaks as made up by the government based on hidden agendas. Research shows that, across contexts and diseases, lack of compliance with public health interventions is often connected with lack of trust in authorities [2,4]. These kinds of suspicions lead to dismissal of risks and disregard of mitigation efforts. In our work in western and central Uganda, Ebola outbreaks have been described as distractions to facilitate government interests, such as control over mining areas or suppression of political opposition. These narratives highlight the importance of trust in governing institutions for successful outbreak response and the limitations of short-sighted and top-down risk communication strategies. Community engagement must build on transparency and trust, and a deeper understanding of livelihood struggles in Ebola-prone settings [2].

Another reason for non-compliance is that local communities perceive that government recommendations on avoiding certain livelihood activities are unrealistic, as alternative livelihood options are not available or not seen as viable [2]. One example is how cross-border trade in bush meat between the DRC and Uganda persists despite repeated warnings about its association with Ebola outbreaks [2]. This underscores the need to adapt outbreak preparedness in dialogue with local communities, not only to ensure trust with authorities, but also to avoid undermining essential livelihood practices. Mitigation strategies should extend beyond emergency medical responses to encompass two-way dialogue with communities to ensure that Ebola response measures are locally understood and trusted, and do not undermine local livelihoods.

### Ebola virus transmission impacted by healthcare- and community dynamics

As expected for an emerging disease, there will be a delay between the index case and the identification of an outbreak. Additionally, high endemic occurrence of differential diagnoses such as malaria, lack of routines to prevent nosocomial infection transmission, and insufficient appreciation of how traditional practices and belief systems shape local responses often lead to misinterpretation or delayed recognition of Ebola symptoms, as observed in the current outbreak. During the time between outbreak and detection, the disease spreads within communities, exposing the public and healthcare workers to risk of infection. The current outbreak in Uganda exemplifies this pattern, having been identified following the burial of a Congolese national who had sought care in Uganda, leading to virus exposure and infection among unsuspecting healthcare workers [6].

Transmission pathways remain consistent across outbreaks, frequently involving avoidable practices such as unprotected contacts between febrile patients and healthcare workers, traditional burials, and resistance to medical isolation [7]. Communities are therefore both the epicentre of transmission and the key to early containment. However, complacency with public health interventions persists [5]. Recent and ongoing events—including escape from Ebola treatment units and attacks on response infrastructure in parts of the DRC—demonstrate that communities have not been sufficiently engaged in preventive initiatives. Past successes demonstrate the importance of inclusive community involvement. The 2014–2016 West Africa Ebola outbreak showed that effective containment required thoughtful long-term engagement with local populations [8].

### Contemporary context: Uganda and the DRC

Uganda and the DRC continue to dominate the landscape of Ebola outbreaks in Africa. The DRC remains affected by prolonged civil unrest and inequality, resulting in widespread poverty, displacement, and fragile health systems [9]. Similarly, the fatal 2000–2001 Ebola outbreak in northern Uganda occurred under conditions of civil unrest. Civil unrest and political instability breed poverty, weaken health systems, and drain public trust. In addition, insecurity limits access to affected populations [10]. Uganda shares borders with the DRC, and regular cross-border movement of people and goods contributes to outbreak spillovers, including in the present outbreak. Consequently, some Ugandans perceive Ebola as an external threat, reducing perceived personal risk and weakening preventive behaviours.

Our findings suggest that the epidemiology and disease impact of Ebola are affected by structural challenges such as poverty, weak governance, and inadequate healthcare systems. In many cases, recurrent Ebola outbreaks are less the primary problem than a symptom of broader systemic inequities [11]. Poverty drives high-risk behaviours such as bushmeat consumption and disregarding public health interventions for survival reasons.

Both Uganda and the DRC also face outbreak governance challenges, including reliance on external funding and limited domestic capacity for outbreak response [12]. Whereas Uganda has shown significant capacities in disease outbreak management [13], its top-down approach to response has impacted how citizens perceive disease outbreaks. The ongoing outbreak clearly demonstrates how weak governance systems can drive outbreaks in unpredictable directions, leading to avoidable mortality and further erosion of public trust.

### Call to action

Addressing Ebola outbreaks sustainably requires comprehensive preparedness and response strategies that account for the social, political, and economic determinants of health. The ongoing outbreak is a critical reminder that efforts must extend beyond emergency response to address underlying inequalities, strengthen local health systems, and build public trust. Key priorities include institutionalizing community engagement to foster transparency and integrating preparedness into routine service delivery.

There is also a need to reframe how socio-cultural practices are labelled in outbreak contexts [14]. Portraying local traditions, livelihoods, and domestic environments as inherently pathogenic risks undermines community dignity and weakens engagement efforts.

**Fig 1:**
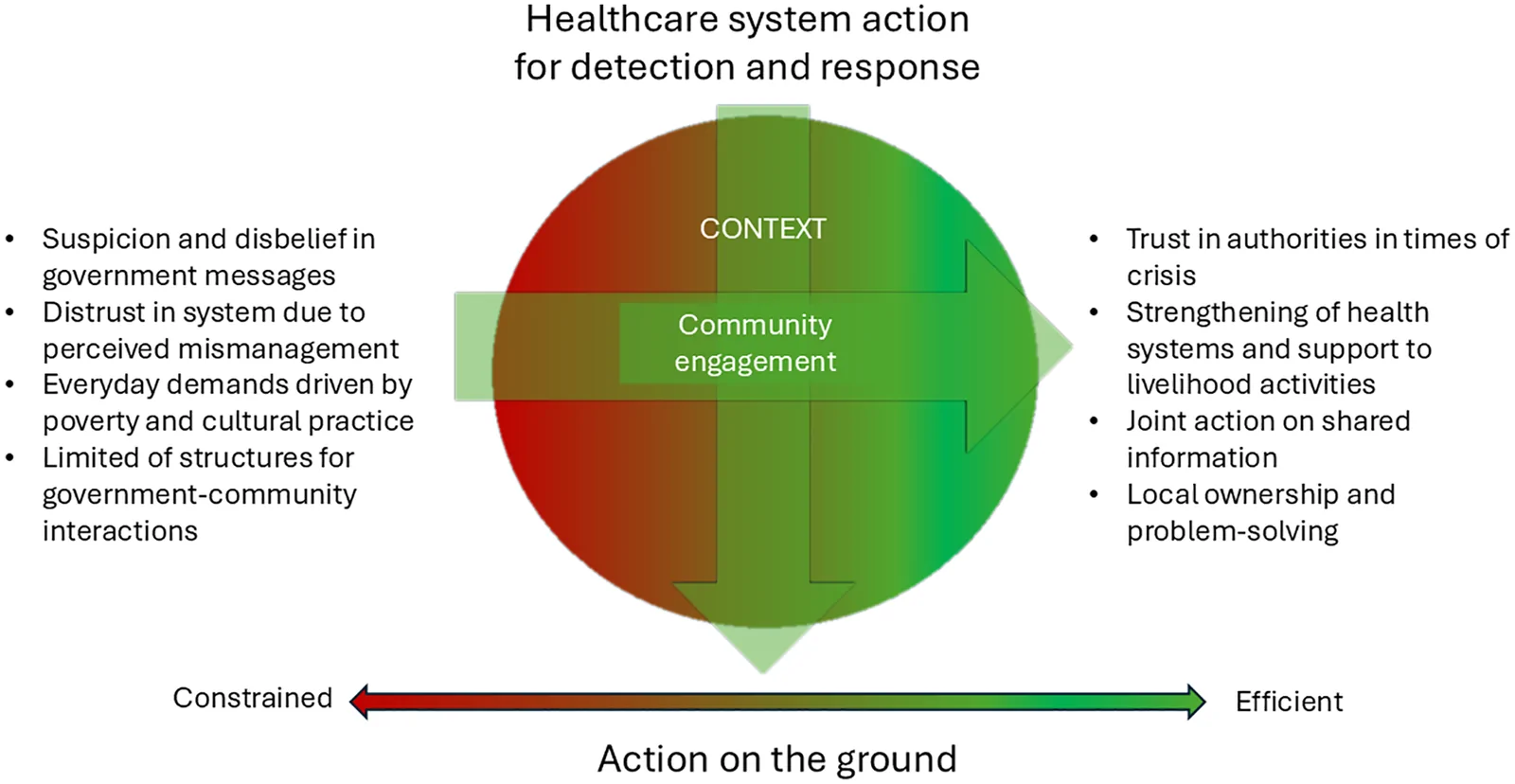
Efficient Ebola response builds on contextually sensitive community engagement.

A more constructive approach involves recognizing and integrating local knowledge systems, addressing structural inequalities, and fostering genuine partnerships between communities and health governance institutions. Only through such holistic and sustained efforts can meaningful preparedness, response, and long-term prevention of Ebola outbreaks—including the current one—be achieved as illustrated in Fig 1 below.
